# Enrollment and Retention Challenges in Early-Phase Intervention Development Studies: The Obesity-Related Behavioral Intervention Trials (ORBIT)

**DOI:** 10.5888/pcd10.130040

**Published:** 2013-03-07

**Authors:** Josephine E. A. Boyington, Susan Czajkowski

**Affiliations:** Author Affiliation: Susan Czajkowski, National Heart Lung and Blood Institute, National Institutes of Health, Bethesda, Maryland 20892.

Recent gains in life expectancy in the United States are threatened by high rates of obesity. Substandard diets and low levels of physical activity have been identified as key determinants of overweight, obesity, and many other chronic conditions (1–3). Fundamental to preventing and treating obesity and its concomitant conditions is the adoption and maintenance of healthful dietary and physical activity habits. However, adherence to obesity interventions, especially over the long term, is low due to patient-, provider-, and systems-related factors (4–6). The problem is particularly evident in vulnerable and underserved populations, including racial/ethnic minorities (7), some low-income groups (7–10), and children (11,12). In light of the broad scope and serious health effects posed by the obesity problem, new and potentially more potent interventions for reducing obesity and obesity-related behaviors are needed, especially in high-risk populations.

Recognizing the need for more effective approaches to reduce obesity, especially in high-risk populations, the National Institutes of Health (NIH) in 2008 issued a funding opportunity announcement (FOA),“Translating Basic Behavioral and Social Science Discoveries into Interventions to Reduce Obesity: Centers for Behavioral Intervention Development (U01)” (3). The FOA solicited research applications to translate insights from the basic and behavioral sciences into clinical, community, and population interventions to reduce obesity (3). Cooperative agreement grants were awarded to 7 project teams and 1 Resource and Coordination Unit (RCU) in September 2009. These research teams, along with representatives from the 5 cosponsoring NIH institutes, centers, and offices (National Heart, Lung, and Blood Institute; National Cancer Institute; National Institute of Diabetes and Digestive and Kidney Diseases; National Institute of Child Health and Human Development; and the NIH Office of Behavioral and Social Science Research) constitute the Obesity Related Behavioral Intervention Trials (ORBIT) initiative (www.nihorbit.org).

The primary goal of the ORBIT initiative is to use the knowledge, skills, and efforts of diverse teams of basic and applied behavioral science researchers in the development of effective obesity prevention and obesity reduction strategies. Targeted groups include high-risk populations, such as black and Hispanic adolescents and adults, premenopausal women, and low-income pregnant women. All ORBIT projects follow a pre-specified, 3-phase approach for intervention development. Research teams first conduct qualitative, observational, and laboratory studies to clarify and refine insights from basic behavioral or social science research to support the development of components of dietary or physical activity interventions. This initial phase is followed by “proof of concept” and small-scale efficacy studies to characterize and provide preliminary efficacy testing of the proposed interventions. The final phase involves pilot studies to refine the interventions, test their feasibility, and obtain information to guide the planning and design of randomized controlled trials. All ORBIT studies have completed at least 1 of the first 2 phases of the intervention development process.

This progressive, phased approach is intended to produce a set of innovative, well-defined, and effective obesity-related interventions that are ready for efficacy and effectiveness testing. The approach has also proven useful in the early identification of recruitment and retention challenges, potentially leading to an improved ability for eventual translation of these interventions into real-world settings. The studies highlighted here exemplify some of the unique challenges faced and attendant solutions engaged in the process of developing obesity-related behavioral interventions.

In the first project — the Maternal Adiposity, Metabolism and Stress (MAMAS) Study (13) — researchers from the University of California, San Francisco, are developing intervention strategies to reduce stress-induced, nonhomeostatic eating (eating reflexively in response to factors other than caloric need or hunger) in low-income pregnant women, focusing on the reward and stress response systems that may influence eating behaviors and abdominal fat deposition. This project required the recruitment of low-income women at a prespecified gestational stage, which posed problems for recruiting adequate numbers of participants. In response, the investigators developed and implemented a set of innovative strategies that are outlined in detail in “Recruitment and Retention of Pregnant Women for a Behavioral Intervention: the Maternal Adiposity, Metabolism, and Stress (MAMAS) Study” (13). For example, investigators gave timely and detailed feedback to their recruitment sources about referral yields. This feedback supports future recruitment efforts by allowing the identification and recognition of high-yield sources and by informing low-yield sources of opportunities for improvement or a change in strategy.

The Small Changes and Lasting Effects (SCALE) project (14), conducted by researchers at Weill Cornell Medical College, aims to refine and pilot test a “small change” eating and physical activity intervention on eating behaviors and weight loss in black and Latino adults in 3 different settings: individual, family, and faith-based settings in Harlem and the South Bronx. In the accompanying article, “Developing Faith-Based Research Partnerships: Recruitment, and Retention Techniques” (14), the authors describe processes that enable successful partnerships with faith-based organizations to support the adequate recruitment and retention of participants and describe considerations for researchers working in settings where faith-based organizations are the main source of research participants.

These 2 ORBIT studies highlight different approaches to the issue of recruitment and retention of research participants in behavioral intervention development studies. Each study encountered unique challenges and applied solutions specific to the research question, population of interest, and research context. The successes described and lessons learned from these formative and early experimental studies convey insights into how characteristics of target populations can influence recruitment outcome (eg, pregnant women in the MAMAS study) and how the settings and organizations in which interventions are to be delivered and from which participants originate (eg, SCALE study) can be best engaged for effective recruitment and retention. The knowledge gained from these studies is primarily useful for improving the quality and practicality of the interventions developed by the ORBIT initiative. However, it can also facilitate the use of more effective approaches for recruitment in the later phases of the intervention development process and can aid in the design of randomized clinical trials to test these and similar interventions.
